# Construction and validation of a necroptosis-related lncRNAs prognosis signature of hepatocellular carcinoma

**DOI:** 10.3389/fgene.2022.916024

**Published:** 2022-08-30

**Authors:** YunZhen Peng, GuoJing Wu, Xin Qiu, Yue Luo, YiShu Zou, XueYan Wei, Aimin Li

**Affiliations:** ^1^ Cancer Center, Integrated Hospital of Traditional Chinese Medicine, Southern Medical University, Guangzhou, China; ^2^ Department of Urology, The Third Affiliated Hospital, Southern Medical University, Guangzhou, China

**Keywords:** hepatocellular carcinoma, necroptosis, long non-coding RNA (IncRNA), prognostic model, immunotherapy, prognostic marker genes

## Abstract

**Background:** Immunotherapy has achieved remarkable success in treating advanced liver cancer. Current evidence shows that most of the available immune checkpoint inhibitor (ICB) treatments are suboptimal, and specific markers are needed for patients regarded as good candidates for immunotherapy. Necroptosis, a type of programmed cell death, plays an important role in hepatocellular carcinoma (HCC) progression and outcome. However, studies on the necroptosis-related lncRNA in HCC are scarce. In this view, the present study investigates the link among necroptosis-related lncRNA, prognosis, immune microenvironment, and immunotherapy response.

**Methods:** Gene transcriptome and clinical data were retrieved from The *Cancer* Genome Atlas database. Pearson correlation analysis of necroptosis-related genes was performed to identify necroptosis-related lncRNAs. The Wilcoxon method was used to detect differentially expressed genes, and prognostic relevant lncRNAs were obtained by univariate Cox regression analysis. Gene Ontology and Kyoto Encyclopedia of Genes and Genomes analysis were utilized to perform functional enrichment analysis. Lasso–Cox stepwise regression analysis was employed to calculate risk score, which was involved in analyzing immune cells infiltration, immune checkpoints expression, and predicting immunotherapeutic efficacy. Quantitative RT-PCR (qRT-PCR) was performed to detect the expression pattern of lncRNA in cell lines.

**Results:** The 10 lncRNAs generated in this study were used to create a prognostic risk model for HCC and group patients into groups based on risk. High-risk patients with HCC have a significantly lower OS rate than low-risk patients. Multivariate Cox regression analysis showed that risk score is an independent risk factor for HCC with high accuracy. Patients in the high-risk group exhibited a weaker immune surveillance and higher expression level of immune checkpoint molecules. In terms of drug resistance, patients in the low-risk group were more sensitive to sorafenib. The OS-related nomogram was constructed to verify the accuracy of our model. Finally, quantitative RT-PCR experiments were used to verify the expression patterns of candidate genes.

**Conclusion:** The lncRNA signature established herein, encompassing 10 necroptosis-related lncRNAs, is valuable for survival prediction and holds promise as prognostic markers for HCC.

## Introduction

Hepatocellular carcinoma (HCC) is a health challenge nowadays, which has become the sixth most common malignant tumor and the fourth-leading cause of cancer death worldwide (Parkin et al., 2005). In addition, HCC is a malignant tumor that ranks fourth for incidence and second for mortality in China (Chen et al., 2016; Zhou et al., 2019). Compared with different regions of Europe and the United States, East Asia and Africa have the highest incidence and mortality of liver cancer (Mcglynn et al., 2015). According to the Surveillance Epidemiology and End Result (SEER) report, liver cancer is expected to be the third leading cause of cancer-related death by 2030 (Rahib et al., 2014).

Surgical resection is still the main method for the treatment of early liver cancer. In pursuit of a higher 5-year survival rate and lower perioperative mortality, surgical indications are usually limited to single tumor, a well-preserved liver function, no portal hypertension, and ECOG score of 0 (Zhou et al., 2001). As the standard therapy for the intermediate stage is radiofrequency ablation and transarterial chemoembolization (TACE), HCC has been the most widely used treatment modality for patients with unresectable HCC. However, HCC is a disease with insidious onset, most patients were diagnosed in the advanced stage and lost the opportunity of operation. At this time, molecular targeted therapy, immunotherapy, and other systemic therapies are of particular importance. Immunotherapy for liver cancer is a promising research direction. Over the past few years, immunomodulator, tumor vaccine, and adoptive immunotherapy have been proved to be effective in clinical practice of liver cancer (Hong et al., 2015). The IMbrave150 study confirmed that the combination of atrizumab and bevacizumab markedly improve the overall survival rate of HCC patients, which has been established as the standard first-line regimen for advanced liver cancer (Finn et al., 2020). In the CheckMate 040 trial (El-Khoueiry et al., 2017), nivolumab, a checkpoint inhibitor that blocks programmed cell death protein-1, demonstrated durable responses and prolonged long-term survival, which has been approved by FDA for second-line therapy of HCC. Nevertheless, cancer treatment, particularly immunotherapy, is urgently in need of the identification of useful biomarkers, as only some patients benefit from this treatment.

In recent years, programmed cell death has aroused great interest in the field of cell death. Nowadays, three forms of cell death including apoptosis, necroptosis, and pyroptosis have been intensely studied. Necroptosis, a kind of programmed cell lytic death, was first proposed by Degterev et al. (2005) to describe a regular rather than accidental necrotic cell death, playing a key role in the regulation of tumorigenesis, cancer metastasis, and cancer immunity (Seehawer et al., 2018). The key molecules of necroptosis promoted the metastasis and progression of cancer alone or in combination with other molecules (Mccormick et al., 2016). Necroptosis is usually regarded as a secondary cell death response in which apoptosis was inhibited, mainly mediated by receptor-interacting protein kinase (RIPK)1, RIPK3, and mixed lineage kinase domain-like protein (MLKL). RIPK1 is a key transcription factor that controls cell survival and death (Christofferson et al., 2014; Weinlich and Green, 2014) and mediates RIPK3-dependant necrosis and FADD-dependent apoptosis (Micheau and Tschopp, 2003; Sun et al., 2012). The morphological changes are characterized by rapid swelling of cells and organelles. The pathway of necroptosis is canonically triggered by the ligand-dependent cell surface death receptors, such as Fas, TNF receptor 1 (TNFR1), IFN receptor (IFNR), and Toll-like receptor (TLR). And necroptosis mediated by TNFR1 is the most representative example (Choi et al., 2019). TNFR1 is homotrimerized and recruits the TNFR-associated death domain (TRADD) protein to its cytoplasmic death domain upon binding of TNF (Hsu et al., 1995; Vandevoorde et al., 1997; Chen and Goeddel, 2002; Gaur and Aggarwal, 2003). When FADD is deficient, RIPK1/RIPK3/MLKL forms a complex called complex IIb or a necrosome to induce necroptosis (Cho et al., 2009; He et al., 2009; Zhang et al., 2009; Sun et al., 2012; Zhao et al., 2012). Caspase-8 is the main inhibitor in the process of necroptosis. When FADD and Caspase-8 were recruited into TRADD and RIPK1 to form complex IIa, Caspase-8 was activated (Mak and Yeh, 2002; Wallach et al., 2002). Activated Caspase-8 induces apoptosis by inhibiting the activity of RIPK1 and RIPK3 (Fritsch et al., 2019). The interactions between RIPK1 and RIPK3 lead to auto-phosphorylate and trans-phosphorylate and the assembly of the “necroptosis complex” when Caspase-8 was inhibited or blocked, which further initiates downstream signaling leading to necroptosis (Cho et al., 2009; He et al., 2009; Zhang et al., 2009). RIPK3 plays a key role in the induction of necroptosis. Phosphorylated RIPK3 activates and phosphorylates MLKL after RIPK3 phosphorylation (Sun et al., 2012), phosphorylated MLKL translocated to the cell membrane leading to the formation of MLKL oligomers, causing its rupture and leading to the leakage of cellular contents and release of DAMP, ultimately triggering the body’s immune response (Gong et al., 2017). Increasing evidence suggests that necroptosis is closely associated with carcinogenesis and anti-tumor immunity. Deletion of MLKL in tumor cells reduces spontaneous lung metastases in a breast cancer model (Jiao et al., 2018). PK68, a kind of RIPK1 inhibitor, significantly inhibits metastasis of mouse melanoma and lung cancer cells (Hou et al., 2019). There is growing evidence that lncRNAs are closely related to programmed cell death (PCD), lncRNA TUG1 knockdown promotes apoptosis by regulating the miR-132-3p–SOX4 axis in osteogenic sarcoma cells (Li et al., 2018). lncRNA MALAT1 promotes colorectal cancer cell proliferation and inhibits apoptosis by activating autophagy (Si et al., 2019).

Non-coding RNA (ncRNA) is a class of RNA longer than 200 nucleotides that do not encode proteins, including microRNA and long non-coding RNA (lncRNA). In cancer, lncRNAs functioning through a variety of mechanisms, such as chromatin remodeling, chromatin interaction, ceRNAs, and natural antisense transcripts (Fang and Fullwood, 2016). LncRNA participates in tumorigenesis and development by different mechanisms (Schmitt and Chang, 2016), a study has shown that lncRNA CASC11 interacts with hnRNP-K to activate the WNT/β-catenin pathway, promoting the growth and metastasis of colon cancer (Zhang et al., 2016). LncRNA HOTAIR is able to predict the sensitivity of ovarian cancer patients by two kinds of platinum chemotherapy, guiding clinical decision-making (Teschendorff et al., 2015). In summary, the imbalance of lncRNA affected cell function, promotion of metastasis, and resistance to chemotherapeutic drugs (Brunner et al., 2012).

With the development of genomics, transcriptome, and next-generation sequencing technology in biological research, the prognostic model constructed by specific gene clusters showed unique advantages in predicting the prognosis of cancer patients and the efficacy of immunotherapy. For example, a prognostic signature based on seven autophagy-related genes is eligible to act as an independent prognostic indicator of poor prognosis (Wang et al., 2021); m6A-associated regulators play a key role in HCC prognosis, tumor microenvironment, and drug resistance (Jin et al., 2021). However, no such study in necroptosis-related lncRNA about HCC has been published yet. Therefore, more studies are needed to explore the prognostic value of necroptosis-related lncRNA in HCC. In this study, we use bioinformatic methods to identify prognostic necroptosis-related lncRNA based on TCGA public database, constructing a prognostic model to verify the role of necroptosis-related lncRNA in HCC. We aimed to identify suitable candidates for immunotherapy and evaluate disease prognosis to better guide therapeutic decisions.

## Methods

### Data acquisition and preprocessing

**FIGURE 1:**
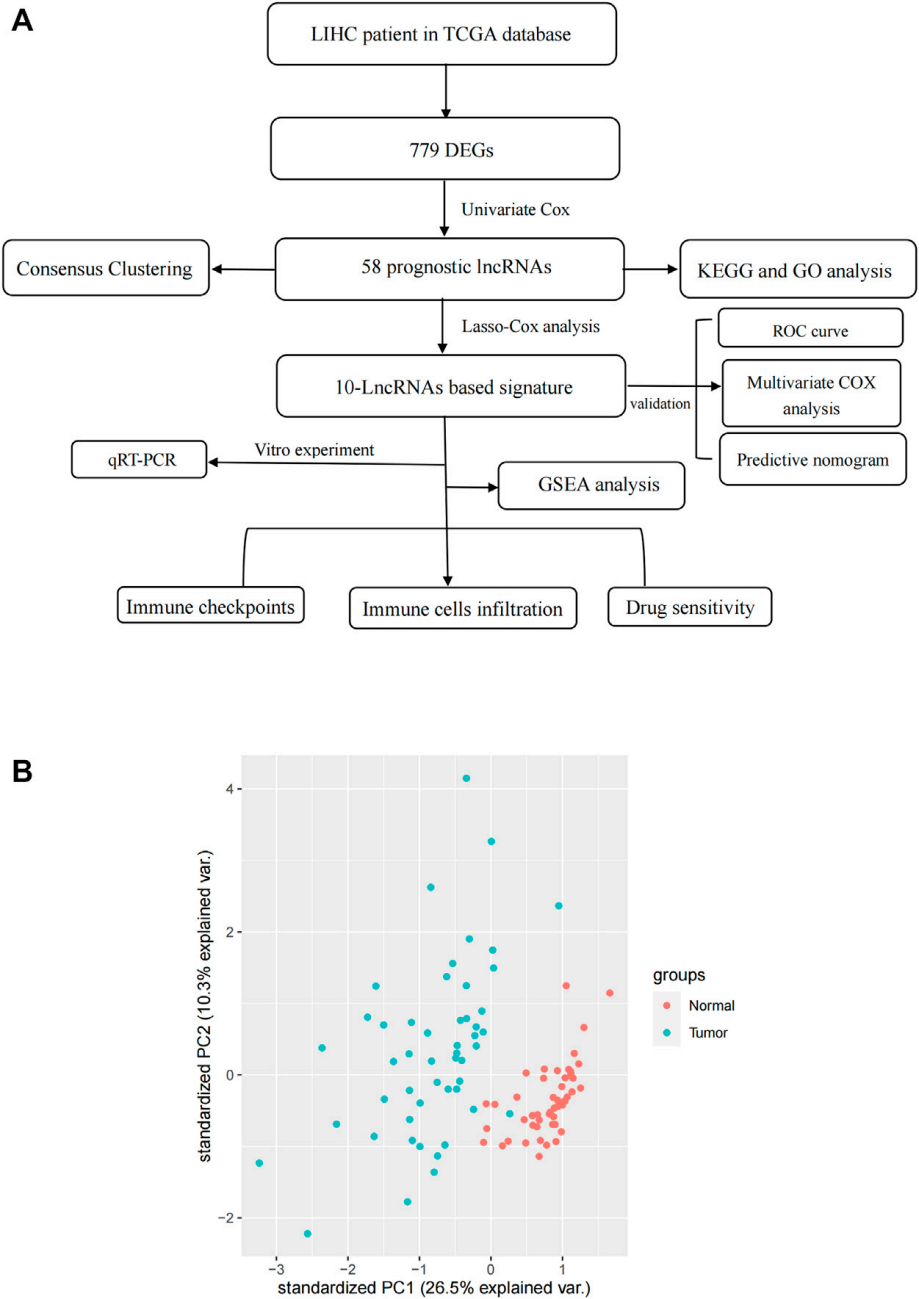
**(A)** Study Flow Chart. **(B)** a PCA plot for RNA-seq data.

We downloaded transcriptome data (TCGA-LIHC-FPKM) and corresponding clinicopathological information of 374 HCC samples and 50 normal samples from The *Cancer* Genome Atlas (TCGA, https://portal.gdc.cancer.gov/). The transcriptome data were converted from FPKM (Fragments Per Kilobase of transcript per Million mapped reads) format to TPM (Transcripts Per Kilobase Million) format by a Perl tool, the human gene annotation file was downloaded from Ensemble database (http://ensemblgenomes.org/). A PCA plot of RNA-sequence data was constructed by “tidyverse” and “dplyr” packages, visualization was prepared in “ggbiplot” package. In this calculation, we included 374 HCC samples and 50 normal samples, 1000 RNAs were detected randomly during this process. One can see that the clustering and integrity of RNA-sequence data were excellent (Figure 1B). Clinical data were preprocessed as follows: Stage IIIA, IIIB, and IIIC were merged into stage III, stage IVA and IVB were merged into stage IV. Samples missing overall survival (OS) data were deleted, finally resulting in a total of 376 samples for survival analysis. The inclusion criteria of this study are as follows: 1) primary hepatocellular carcinoma confirmed by pathology; 2) all patients were available of their survival data; 3) there was complete data for all the samples. All data were downloaded from the public databases; hence, it was not required to obtain additional ethical approval for our study. A total of 67 necroptosis-related genes were used to perform analysis. Spearman’s analysis was conducted to identify necroptosis-related lncRNAs with the identification criteria of Spearman’s correlation coefficient with an absolute value of >0.4 and *p* < 0.001.

### Identification of DEGs

The “bioconductor limma” package was applied to identify differentially expressed genes (DEGs) between cancer and normal samples, |log2foldchange|>1 and the false discovery rate (FDR) < 0.05 were the cut-off criteria for DEGs. The volcano plot was further figured to show the DEGs of TCGA. The results were presented as heatmap and volcano map.

### Identification of prognostic-related lncRNAs (PRlncRNAs)

Univariate Cox regression analysis was performed on DEGs of the TCGA cohort to obtain prognostic-related lncRNA (PRLncRNA). The relationship between PRLncRNA and necroptosis-related genes was visualized by the “ggalluvial” package.

### Gene ontology (GO) and kyoto encyclopedia of genes and genomes (KEGG) analysis

GO and KEGG analyses of PRLncRNAs were performed by R package “ClusterProfiler.” Enrichment analysis was conducted by using the functions “enrichGO” and “enrichKEGG.” The bubble diagram was generated by R packages “ggplot2,” “enrichplot,” and “GOplot.” The FDR value of *p* < 0.05 was considered significantly enriched terms.

### Consensus clustering analysis

Consensus clustering is a resampling-based clustering algorithm, which quantifies the consensus between several clustering iterations and provides means to estimate the number of clusters that best fit data. In this study, data sets were clustered by k-means with k from two to nine by using the “ConsensusClusterPlus” package. Survival analysis between clusters was conducted using the log-rank test and Kaplan– Meier curve, and these analyses were performed with the “ggsurvplot” function in the survival R package. Correlations between clusters and clinical features were performed through the chi-square test. *p < 0.05, **p < 0.01, and ***p < 0.001.

### Construction and validation of the prognostic risk signature

Least absolute shrinkage and selection operator (LASSO)-Cox regression analysis of overall survival (OS) with a 10-fold cross-validation was performed to screen for necroptosis-related lncRNAs with prognostic values, which may preserve valuable variables and avoid overfitting and delete highly correlated genes. The optimal coefficient was determined and the deviance likelihood was calculated by the “glmnet” package (Supplementary Figure S3A). The optimal value of the shrinkage parameter (lambda) was the minimum as selected by 10-fold cross-validation (Supplementary Figure S3B). The risk score of each sample was calculated based on the LASSO-Cox regression co-efficiency through the following formula: Risk score = β1*Exp1 + β2*Exp2 + βi*Expi, where β represents the regression coefficient and Exp represents the gene expression value (Supplementary Table S2).

A total of 370 patients with HCC were randomly divided into training group (n = 186) and verification group (n = 184) in the ratio of 1:1. There was no significant difference in clinical covariables, including age, molecular subtype, grade, TNM stage, and survival status between the two groups (Table 1). According to the median risk score, each group was divided into high-risk subgroup and low-risk subgroup. Univariate Cox regression was used to screen the prognostic variables significantly related to OS in the training group, verification group, and the whole cohort. Afterwards, statistically significant variables were included in multivariate COX regression analysis. Results were reported as hazard ratio (HR) with 95% confidence interval (CI). The sensitivity and specificity of the model for predicting the 1-, 3-, and 5-year survival rates of HCC patients were evaluated by an ROC curve constructed by the “timeROC” package. In addition, to further compare the predictive efficacy of risk scores compared with other clinical features, we constructed an ROC curve for predicting 3-year OS in HCC patients and calculated the AUC.

**TABLE 1:** Clinicopathlogical features of high- and low-risk group.

Variables			Test cohort	Training cohort	*p* Value
Number of patients			190	187	
Age	≤65		120	115	0.873
	>65		70	71	
	Unknown		1	0	
Gender	Female		64	58	0.661
	Male		127	128	
Grade	G1		27	28	0.227
	G2		95	85	
	G3		56	68	
	G4		10	3	
	Unknown		3	2	
T stage	T1		102	83	0.480
	T2		43	52	
	T3		38	43	
	T4		7	6	
	Unknown		1	2	
M stage	M0		139	133	0.931
	M1		2	2	
	unknown		50	51	
N stage	N0		133	124	0.530
	N1		1	3	
	Unknown		57	59	
TNM stage	I		98	77	0.412
	II		41	46	
	III		39	47	
	IV		2	3	
	unknown		11	13	

### Construction and validation of a predictive nomogram

Based on the statistically significant factors of multivariate Cox regression analysis, a prognostic nomogram was constructed to predict the survival probability of patients diagnosed with HCC. Samples with missing entries were removed from this analysis, a total of 240 patients were included in this analysis, the overall survival time of 1, 3, and 5 years were used as the end point. R package “RMS” was used to perform this process, the predictive accuracy of this model was assessed by employing concordance index (C-index). The 95% CI = (C-index ± 1.96) *SE, the predictive accuracy of the proposed model was reflected by the overlap between the calibration curve and the diagonal.

### Tumor immune cells infiltration (TICL) and correlation analysis between high- and low-risk subgroups

In order to explore the lncRNAs signature whether plays a key role in immune infiltration of HCC, we calculated the abundance of 22 types of infiltrating immune cells in each sample by CIBERSORT algorithm. The differential infiltration levels of TICLs between high- and low-risk groups were calculated by the Wilcox test, violin plots were generated using the “vioplot” package. Single-sample gene-set enrichment analysis (ssGSEA) was conducted by the “GSVA” package. Correlations was analyzed by Spearman’s non-parametric test. *p < 0.05, **p < 0.01, and ***p < 0.001.

### Estimation of tumor immune microenvironment (TIME)

The immune score and stromal score of each sample were estimated using the ESTIMATE algorithm by “estimate” package. Results were presented in violin and boxplot plots, methods the same as before.

### Differential expression of immune checkpoint molecules between high- and low-risk subgroups and correlation analysis

The differential expression of programmed death receptor 1 (PD-1, also known as PDCD1), programmed death ligand 1 (PD-L1, also known as CD274), cytotoxic T lymphocyte antigen 4 (CTLA-4), and indoleamine-1 (IDO-1) in high- and low-risk subgroup were analyzed by the Wilcox test. Spearman’s method was carried out for correlation analysis between immune checkpoint molecules and risk score. The “ggExtra” package, “ggplot2″ package, and “ggpubr” package of R software participated in the production of box chart and scatter chart.

### Gene set enrichment analysis (GSEA)

Gene set “c2.cp. kegg. v7.5.1. symbols. Gmt” (downloaded from http://www.gsea-msigdb.org/gsea/downloads.jsp) acted as an input preparation file in GSEA analysis performed by “clusterprofiler” package (*p* value cutoff = 0.01 and q value cutoff = 0.05). |NES| > 1, adjust *p*-value<0.05 and FDR<0.25 were considered to be a significant enrichment item. Enrichment plots were displayed with the “enrichplot” package.

### Evaluation of sorafenib sensitivity in high- and low-risk subgroups

The R package “pRRophetic” was used for predicting the semi-inhibitory concentration (IC50) of patients in high- and low-risk groups treated with sorafenib. The lower the number, the stronger the binding affinity and the higher the drug sensitivity.

### Cell culture

Human hepatic cell line L-O2 and four human HCC cell lines (HepG2 cell, Hep3B, LM3, and Huh7 cells) were flashed frozen in liquid nitrogen with 2 ml tubes and stored at −80°C. All cell lines were cultured in 10% fetal bovine serum (FBS; Invitrogen, Carlsbad, California, United States) in DMEM medium. All cell lines grew in a humid environment of 37°C, 5% CO2, 99% relative humidity and did not contain antibiotics. The cells were subcultured at a ratio of 1:2 or 1:3 when they reached 80% confluence.

### RNA extraction and quantitative real-time PCR (qRT–PCR)

TRIzol reagent kit (Invitrogen) was used to extract the total RNA from logarithmic growth cells. cDNA was synthesized using PrimeScript RT reagent kit (Takara Biotechnology, Dalian, China). Quantitative real-time PCR analysis was conducted using TB Green Premix Ex Taq II kit (Takara Biotechnology, Dalian, China) according to the instructions, three replicates were set in each well. All operations were carried out on ice. The 2^−ΔΔCt^ method was used for quantitative PCR analysis. The primers were designed using the NCBI primer-BLAST tool (https://www.ncbi.nlm.nih.gov/). The sequences of primers were showed in supplement Table S3, ACTB was used as reference genes (Table 2).

**TABLE 2:** Details of 58 PRLncRNAs.

Gene	Coef
AL031985.3	0.351023768463183
SREBF2-AS1	0.0386144854777114
ZFPM2-AS1	0.0102274968460904
KDM4A-AS1	0.114565023598859
AC026412.3	0.167864746612448
AC145207.5	0.0920876812602594
DUXAP8	0.46617052054734
LINC01224	0.181433562593978
AC099850.4	0.00947324010819726
MKLN1-AS	0.262823244119484

### Statistical analysis

All bioinformatics analyses were performed by R 4.0.2, *p* < 0.05 was considered significant in all analysis, unless indicated otherwise. KM curve and log-rank test were used for survival analysis. All continuous variables were tested by Wilcoxon test or independent sample *t*-test, all qualitative data were compared by chi-square test, all tests were conducted as two-sided tests. Cox proportional hazard regression analysis was used to determine the independent predictors of HCC patients. SPSS (IBMSPSS25.0, SPSS Inc.) and Graphpad prism 8 (GraphPad Software, SanDiego, CA, United States) were used for statistical analysis. *In vitro* experiment, each group repeated at least three independent experiments (Table 3).

**TABLE 3:** q-PCR primer sequence.

Gene	HR	HR.95 L	HR.95H	p
AC009005.1	1.17110969344691	1.07429391453767	1.27665054742077	0.000333551251369458
AC018690.1	2.10384322910643	1.43080767874763	3.09346699657856	0.000156073183457983
LUCAT1	1.18189988087072	1.09837739997351	1.27177355291169	7.84559781562504e-06
AL117336.2	1.56655126336053	1.30183152048208	1.88510020085217	2.00524463719125e-06
AC010864.1	5.49638443883633	2.10858881432178	14.3272323623699	0.000490074433174014
AL031985.3	1.83467899783164	1.49647955721917	2.24931039575273	5.29468369207119e-09
SREBF2-AS1	1.45642902550033	1.16706193665077	1.81754321660702	0.000877895411678177
AC034229.4	1.97472355301551	1.40030533397342	2.78477344635196	0.000104567297347242
AC074117.1	1.46708753382555	1.19327667886859	1.80372739199687	0.000276371482958239
THUMPD3-AS1	1.36425983075505	1.16119422832644	1.60283683849706	0.00015843033051424
AC026401.3	1.08418641157238	1.03570125862827	1.13494134070575	0.00053473105754009
AC125437.1	2.5548070290898	1.58555156851862	4.11657311277797	0.000116331481881563
AL049840.4	2.0302971636625	1.36352982623686	3.02311434151197	0.000489306477985457
MED8-AS1	2.38264064141742	1.47164608808717	3.85756906642744	0.000412937020718624
AC131009.1	1.81743270484487	1.39795412601752	2.36278256572658	8.11473526933209e-06
BBOX1-AS1	1.26356802863024	1.10450153968093	1.44554272277225	0.000654722408806783
ZFPM2-AS1	1.09331071475861	1.05267666559006	1.13551326639881	3.90143830862613e-06
ZEB1-AS1	1.45367588907736	1.16714497469377	1.81054936302091	0.000838235675022376
DDX11-AS1	4.30711049547743	2.04855911473802	9.05573126339791	0.000117458622091245
MCM3AP-AS1	8.94040972369425	2.69227895856596	29.688946523618	0.000347151833494758
ZNF337-AS1	2.73367363684378	1.52991790481875	4.88455722312761	0.00068428899137241
AC012073.1	1.40901745291795	1.22743803553936	1.61745858050992	1.10884707035332e-06
LINC01094	2.00660050130517	1.39264200898897	2.89122800105769	0.000186022552058713
LINC01138	1.44794724338591	1.22020644496107	1.71819385833168	2.24120525795787e-05
AC100872.2	1.52447962710877	1.24327474005833	1.86928766312799	5.05641699168209e-05
AL355574.1	1.28242891205497	1.11158436232424	1.47953135202056	0.00064918759120028
KDM4A-AS1	2.86701953213825	1.90687848154453	4.3106055667503	4.14527699473686e-07
AC016394.2	1.57882015234359	1.23080436529767	2.02523905807191	0.000324985331627426
AC026412.3	15.090021090766	4.61045168516158	49.3896806797968	7.24800949113819e-06
AL365203.2	1.13673308566103	1.07468028025401	1.20236886428308	7.6533988362139e-06
AC145207.5	1.76721729739874	1.3628688724496	2.29153151807772	1.74349659249233e-05
AC107068.1	2.10949184257841	1.44055306556969	3.08906068111073	0.000125211421420502
AC012467.2	1.49459418738293	1.18257552681207	1.88893794460693	0.00076932764792714
AC025176.1	2.28280817121522	1.48702718115843	3.50445049868376	0.000160460084632489
DUXAP8	4.37169584194494	2.02781234526238	9.42479938004609	0.000167423690730116
TMCC1-AS1	2.56854719180891	1.77460682269685	3.71768810542686	5.72455165204052e-07
NCK1-DT	1.51326486674367	1.20122630200084	1.90636065253186	0.00043798957870755
LINC01224	1.92867470390563	1.44091805772471	2.58153896645535	1.00747168565781e-05
NRAV	1.23212492752684	1.12566307516838	1.3486556239805	5.97410050681252e-06
AL442125.2	3.83473706827137	2.04401302301756	7.19428311717178	2.82689097369676e-05
AP003392.4	7.36176131533239	2.58151629641687	20.9936810157455	0.000188653499478888
MIR210HG	1.14187648047771	1.06700456384948	1.22200217397766	0.000125916300801374
GHRLOS	3.47983906642121	1.79075574511339	6.76210586576923	0.000234274247349297
AC026356.1	4.01051373646868	2.129621086889	7.55262075935777	1.70265345012451e-05
AC099850.4	1.13959993843801	1.08696785648712	1.19478051897969	6.0760619159702e-08
AC026355.2	2.32443180244038	1.66331751099788	3.24831739488801	7.81777059889033e-07
AL928654.1	1.91049713067697	1.31021936911352	2.78579249579746	0.000768120091986322
POLH-AS1	2.56671257723233	1.7123957107321	3.84724944873056	5.00030914523686e-06
C2orf27A	1.67756319510261	1.35037659748986	2.08402476671623	2.9600669059356e-06
SNHG4	1.47583880290837	1.27644304580875	1.706382575644	1.47410333621925e-07
BACE1-AS	1.21074284838357	1.09643625670793	1.33696622666723	0.000157124984312145
SNHG3	1.06757970081289	1.03740139549618	1.09863590172115	7.83216781994623e-06
AC073611.1	1.48893059282573	1.17720442829988	1.88320248969337	0.000896616489881415
SNHG10	1.29463387153636	1.12504691265138	1.48978397476709	0.000312461913317886
AC107959.3	1.38256463352294	1.15262199806244	1.65837973687959	0.00048225465968827
MKLN1-AS	3.36374917116319	2.23555078410734	5.06130684524757	5.91261812769369e-09
AC006252.1	4.77420311932314	1.95702613952842	11.6467608501372	0.000591250187772917
HMGN3-AS1	2.12745977534335	1.39332694836852	3.24840131815699	0.000472264012528417

## Results

### Data acquisition and establishment of co-expression network

**FIGURE 2:**
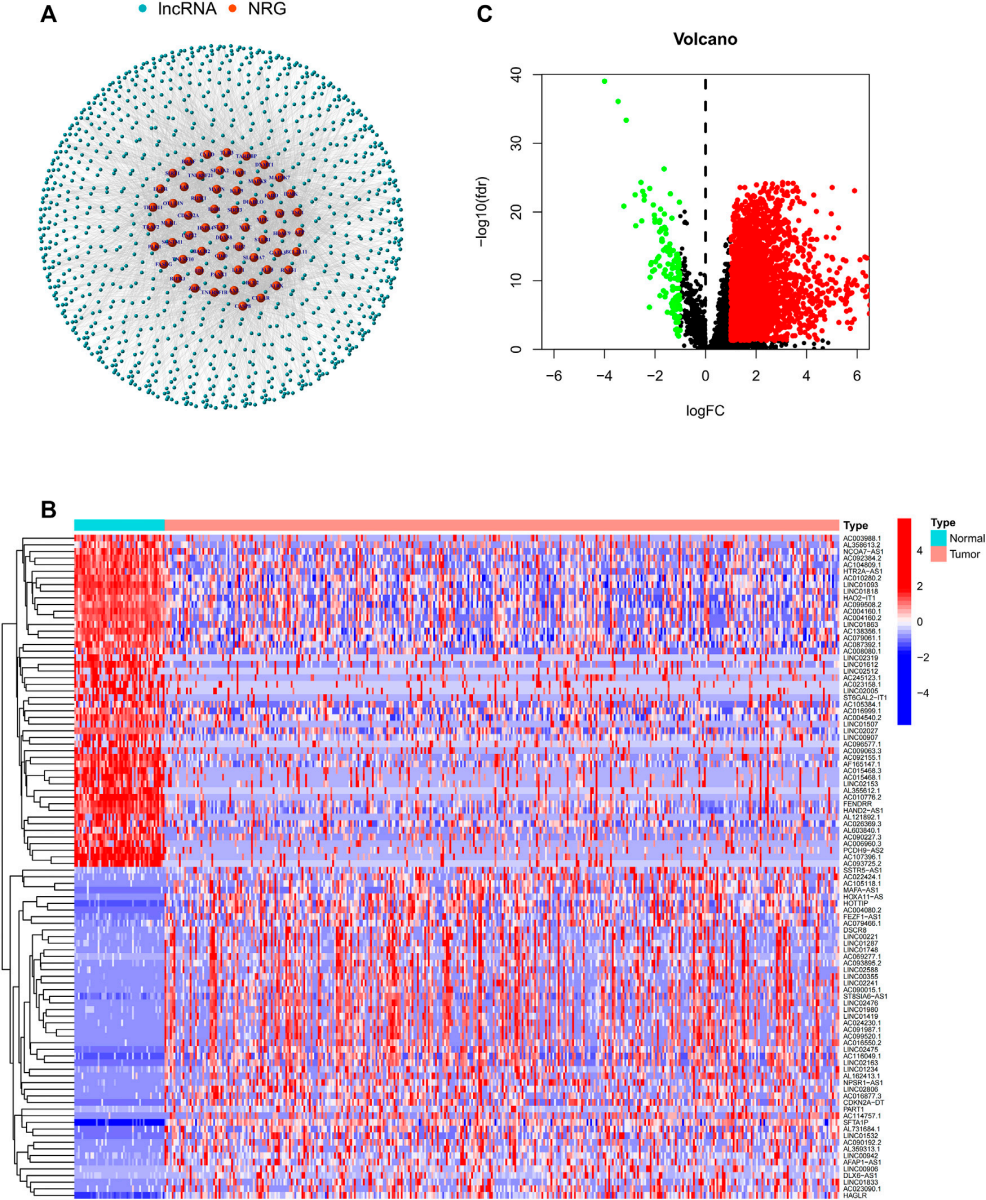
**(A)** Co-expression network between necroptosis-related genes and lncRNAs. **(B)** Differential expression of DEGs in tumor and adjacent normal pairs. **(C)** A volcano map shows DEGs. DEG: differentially expressed genes.

The flow chart of this study is shown in Figure 1. RNA-Seq data of LIHC samples were downloaded from TGCA database. We adopted 67 necroptosis-related genes (FADD, FAS, FASLG, MLKL, RIPK1, RIPK3, TLR3, TNF, TSC1, TRIM11, CASP8, ZBP1, MAPK8, IPMK, ITPK1, SIRT3, MYC, TNFRSF1A, TNFSF10, TNFRSF1B, TRAF2, PANX1, OTULIN, CYLD, USP22, MAP3K7, SQSTM1, STAT3, DIABLO, DNMT1, CFLAR, BRAF, AXL, ID1, CDKN2A, HSPA4, BCL2, STUB1, FLT3, HAT1, SIRT2, SIRT1, PLK1, MPG, BACH2, GATA3, MYCN, ALK, ATRX, TERT, SLC39A7, SPATA2, RNF31, IDH1, IDH2, KLF9, HDAC9, HSP90AA1, LEF1, BNIP3, CD40, BCL2L11, EGFR, DDX58, TARDBP, APP, and TNFRSF21) and 14,086 lncRNA (Downloaded from Ensemble database) in this study. At last, we obtained 1026 necroptosis-related lncRNAs. We established a co-expression network to visualize the relationship between necroptosis-related lncRNAs and 67 necroptosis-related genes (Figure 2A).

### Identification of DEGs

In this study, a total of 779 DEGs were obtained through the calculation by the “limma” package. Because of the excessive number of genes with significant differences, only the top 50 lncRNAs with significant differences are displayed in Figure 2A. As shown in Figure 2B, most of the DEGs are highly expressed in HCC and differentially expressed lncRNAs were shown through volcano plot filtering (Figure 2C).

### GO and KEGG analysis of PRlncRNAs

**FIGURE 3:**
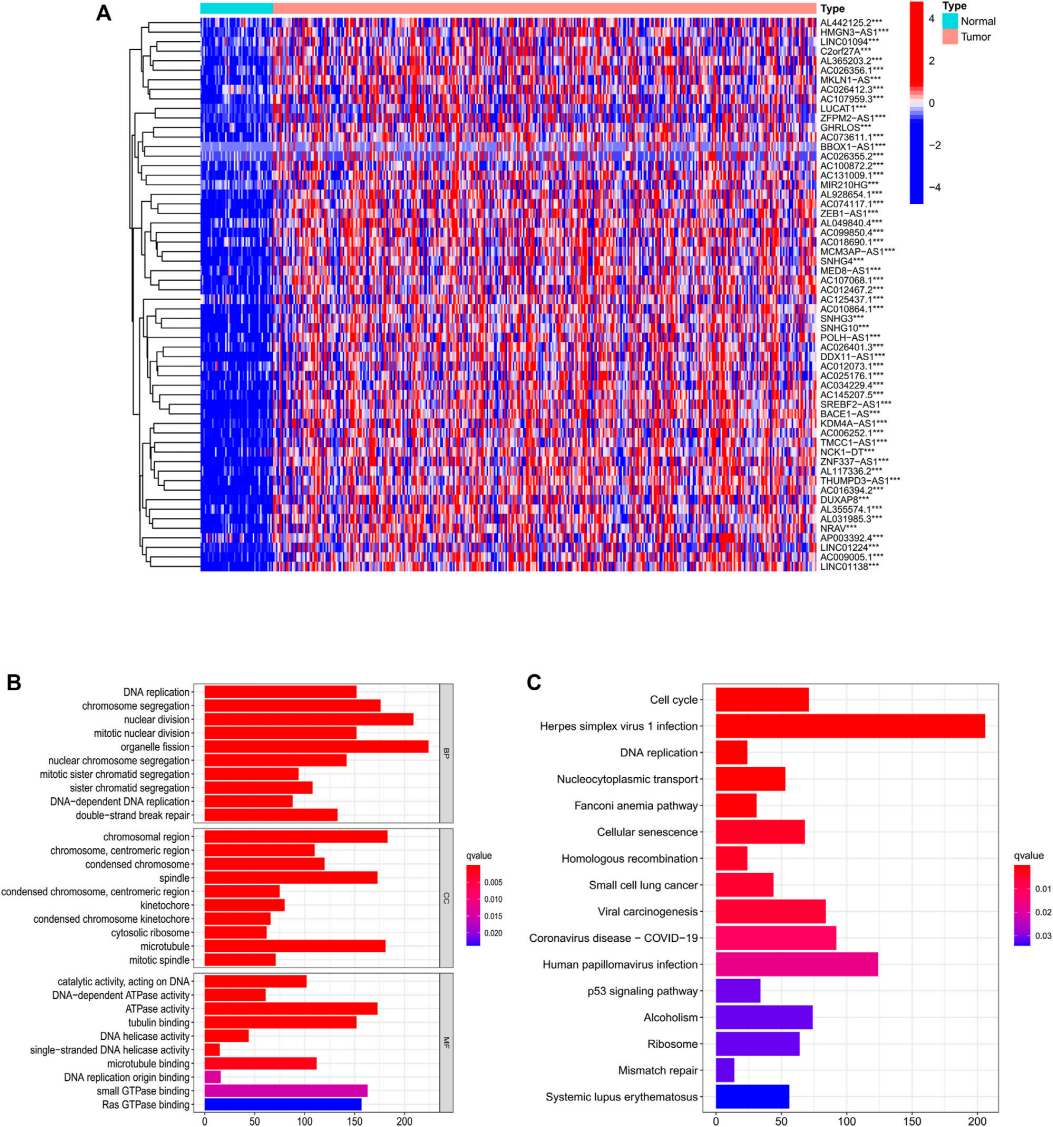
Identification of PRlncRNAs. **(A)** Differential expression of PRlncRNAs in tumor and adjacent normal pairs. **(B–C)** GO and KEGG enrichment analysis results. PRlncRNAs: prognostic-related lncRNA; GO: Gene Ontology; KEGG: Kyoto Encyclopedia of Genes and Genomes.

Among 779 DEGs, 58 necroptosis-related lncRNAs (PRlncRNAs) were obtained by univariate Cox regression analysis (Supplementary Table S1). As shown, PRlncRNAs were highly expressed in HCC samples (Figure 3A). In order to further explore the potential biological pathways by which these genes affect the prognosis of HCC patients, we carried out GO and KEGG analysis. Biological process (BP), molecular function (MF), and cellular component (CC) were the three categories of GO analysis. The biological process analysis revealed that “organelle fission (GO:0048285),” “nuclear division (GO:0007088),” “chromosome segregation (GO:0051983),” and “DNA replication (GO:0033260)” were the most abundant terms in the BP category. At the level of MF aspect, ATPase (GO:1904949), small GTPase binding (GO:0031267), and Ras GTPase binding (GO:0017016) were significantly enriched. The cellular component (CC) indicated that these genes were predominantly located in the chromosomal region (GO:0098687), microtubule (GO:0005874), and spindle (GO:0005819) (Figure 3B). KEGG analysis showed that PRlncRNAs mainly localized to the Herpes simplex virus 1 infection pathway (hsa05168), human papillomavirus (HPV) infection pathway (hsa05165), coronavirus disease—COVID-19 pathway (hsa05171), viral carcinogenesis pathway (hsa05203), and cell cycle (hsa04110) pathway (Figure 3C).

### Development and validation of prognostic PRlncRNA signature

**FIGURE 4:**
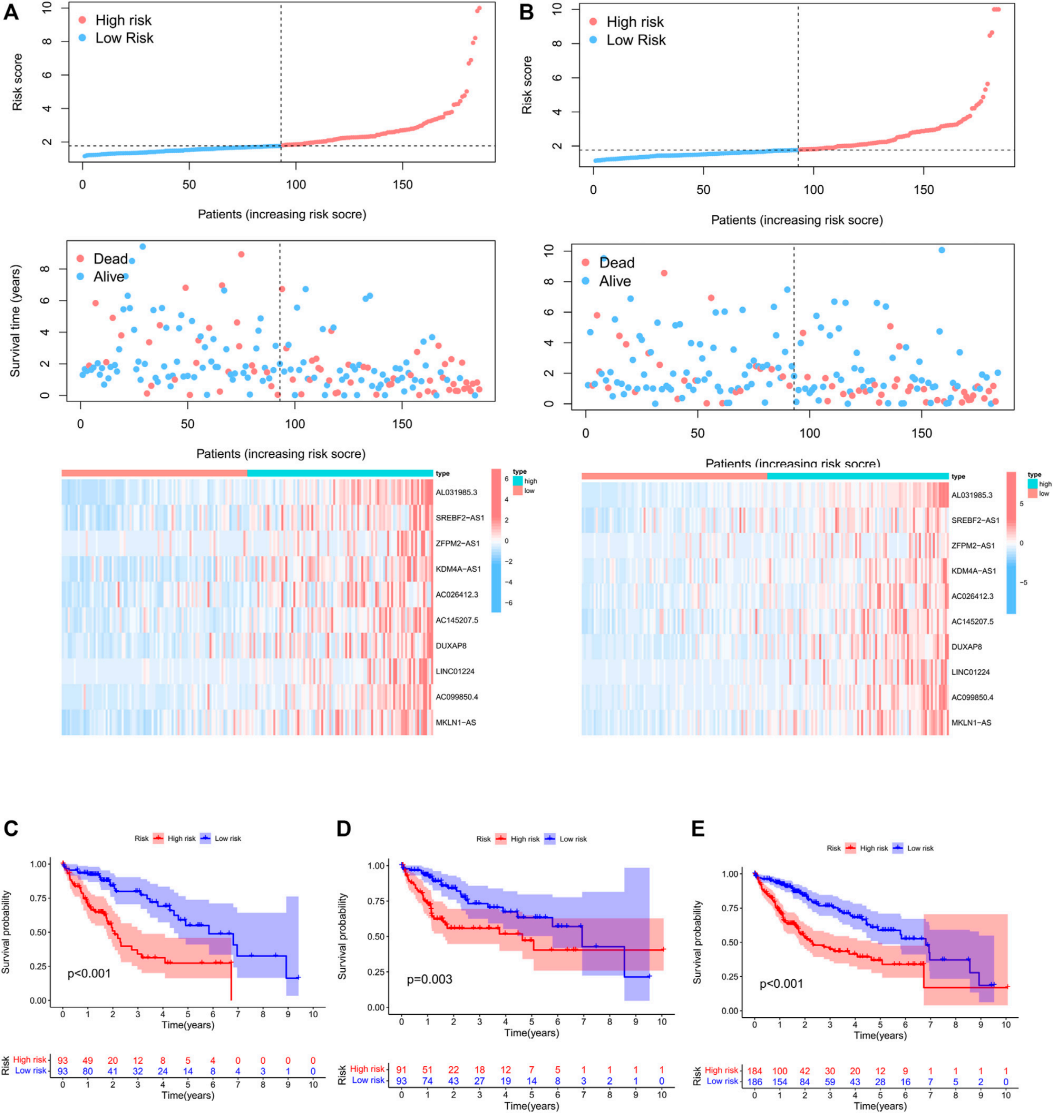
Construction and validation of prognostic PRlncRNAs signatures in TCGA cohorts. **(A)** Risk factor curve, survival status of patients, and heatmap of the differential expression of 10 PRlncRNAs in the TCGA training cohort. **(B)** Risk factor curve, survival status of patients, and heatmap of the differential expression of six PRlncRNAs in the TCGA test cohort. **(C–E)** Kaplan–Meier survival curves of the TCGA training cohort, internal validation cohort, and whole cohort.

To further evaluate the prognostic value of necroptosis-related lncRNAs, based on 58 candidate PRlncRNAs, we applied LASSO-COX stepwise regression analysis to screen 10 PRlncRNAs which were most correlated to the HCC patient’s OS. Based on this, we constructed a risk signature based on 10 lncRNAs. All samples were divided into training group (n = 186) and internal verification group (n = 184) at about a ratio of 1:1 randomly, there were no significant differences in age, gender, stage, grade, T stage, N stage, and M stage between the experimental group and the verification group (Table 1). According to the median of risk score, each group was then divided into high-risk subgroup and low-risk subgroup. Through LASSO-Cox stepwise regression analysis, AL031985.3, SREBF2-AS1, ZFPM2-AS1, KDM4A-AS1, AC026412.3, AC145207.5, DUXAP8, LINC01224, AC099850.4, MKLN1-AS were considered to be the best predictors (Table 4). The coefficient was calculated by LASSO-Cox regression. The risk score formula was as follows: AL031985.3*0.351023768463183 + SREBF2-AS1*0.0386144854777114 + ZFPM2-AS1*0.0102274968460904 + KDM4A-AS1*0.114565023598859 + AC026412.3*0.167864746612448 + AC145207.5*0.0920876812602594 + DUXAP8*0.46617052054734 + LINC01224*0.181433562593978 + AC099850.4*0.00947324010819726 + MKLN1-AS*0.262823244119484. Compared with the low-risk subgroup, the higher the mortality of the HCC patients in the high-risk group, the shorter the OS. Also, the expression of lncRNAs was positively correlated with the risk score (Figure 4A,B).

**TABLE 4:** LncRNAs constituting a prognostic model.

Gene	Forward primer (5’→3′)	Reverse primer (5’→3′)
ACTB	CCTCGCCTTTGCCGATCC	CGC​GGC​GAT​ATC​ATC​ATC​C
SREBF2-AS1	CAA​CGG​GGA​TGT​AGC​CAT​CA	ATC​TCT​CCG​GGA​TGG​AGA​CC
ZFPM2-AS1	TGG​CAG​AGT​TGC​ACA​GAA​GA	ACC​ACT​CAC​ACT​TTC​ATC​GCT
KDM4A-AS1	GAG​GGT​GAA​AGG​AAC​GTC​CA	AAG​TAC​TTT​GCC​AGG​TCC​CA
AC026412.3	GCG​TAG​ATC​CCT​TTG​GCT​CA	GAC​CTC​CAT​TGA​AGG​GCT​CA
AC145207.5	GAC​TGG​CCA​AGC​ATT​TGG​TG	TCT​GGC​CTA​CCT​TAG​GCT​ACA​T
DUXAP8	CAC​CAG​CCT​CAC​TAG​CAC​TC	ACA​CCC​GGC​CAA​GTT​CTT​TA
LINC01224	ACA​GGA​TTG​TTA​ATC​TCA​TCT​TGG​A	TCA​GGT​TCT​ACA​CAG​AGG​CA
AL031985.3	ACA​ACA​CAT​CAA​GAG​GCC​CA	TTC​CCT​GCC​TGA​GTA​TGG​CT
MKLN1-AS	TCT​CTG​AAA​GCA​GCG​CTT​GG	AGT​CCT​CAA​GGT​ATG​GGG​GA

Kaplan–Meier survival analysis showed that the total survival time of HCC patients in the high-risk group was significantly shorter than that in the low-risk cohort, both in the training cohort, validation cohort, and whole cohort (Figure 4C–E), suggesting that the prognostic risk score is a valuable predictor of prognosis.

**FIGURE 5:**
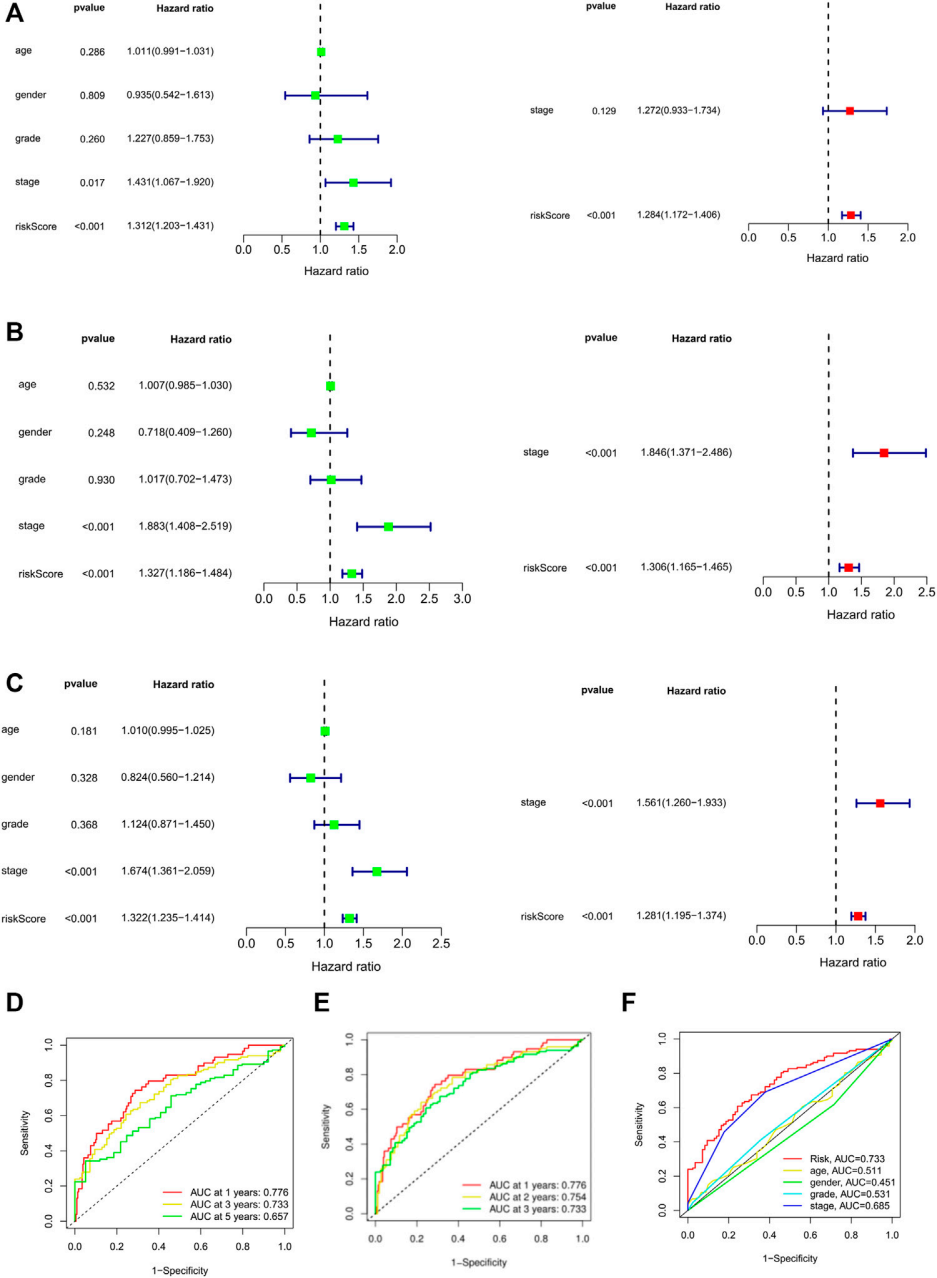
Univariate and multivariate Cox regression model for prognostic risk. **(A–C)** The univariate and multivariate Cox analysis of patients OS-related risks in TCGA training cohort, internal validation cohort, and whole cohort. **(D)** ROC curves of 1, 3, and 5 years based on the 10 PRlncRNAs signatures in the whole cohort. **(E)** ROC curves of 1, 2, and 3 years based on the 10 PRlncRNAs signatures in the TCGA whole cohort. **(F)** ROC curves using multiscale features. OS: overall survival.

In order to further screen factors that can be seen as independent prognostic factors to predict OS in patients with HCC, risk scores combined with common clinical prognostic factors including age, gender, histological grade, and pathological stage were included in the univariate Cox regression analysis. Univariate (HR = 1.31; 95%CI = 1.20–1.43; *p* < 0.001) and multivariate COX (HR = 1.28; 95% CI = 1.17–1.40; *p* < 0.001) regression analyses were conducted in the training cohort, suggesting that prognostic risk score can be seen as an independent factor affecting survival in HCC patients (Figure 5A). Furthermore, risk score and pathological stage were both important independent prognostic factors for OS in multivariate regression analyses of internal validation cohort (Figure 5B) and whole cohort (Figure 5C). In any case, it is certain that the prognostic risk score based on necroptosis-related lncRNAs was capable of predicting the OS of HCC patients independent of other clinical factors.

The ROC curve showed that the area under the receiver operating curve (AUC) of predicting the OS of 1-, 3-, and 5-year HCC patients was 0.776, 0.773, and 0.657, respectively (Figure 5D). Because of the low 5-year survival rate of HCC patients, we chose to predict the 1- (AUC = 0.776), 2- (AUC = 0.754), and 3-year (AUC = 0.733) OS for further analysis (Figure 5E). Also, compared with other clinicopathological features (AUC of 3-year < 0.5), the prognostic risk score (AUC of 3-year = 0.733) showed a superior prediction ability (Figure 5F). ROC analysis showed that the accuracy of prognostic risk score was higher than that of other clinicopathological features, indicating that our risk signature showed excellent predictive performance.

### Subgroup analysis

**FIGURE 6:**
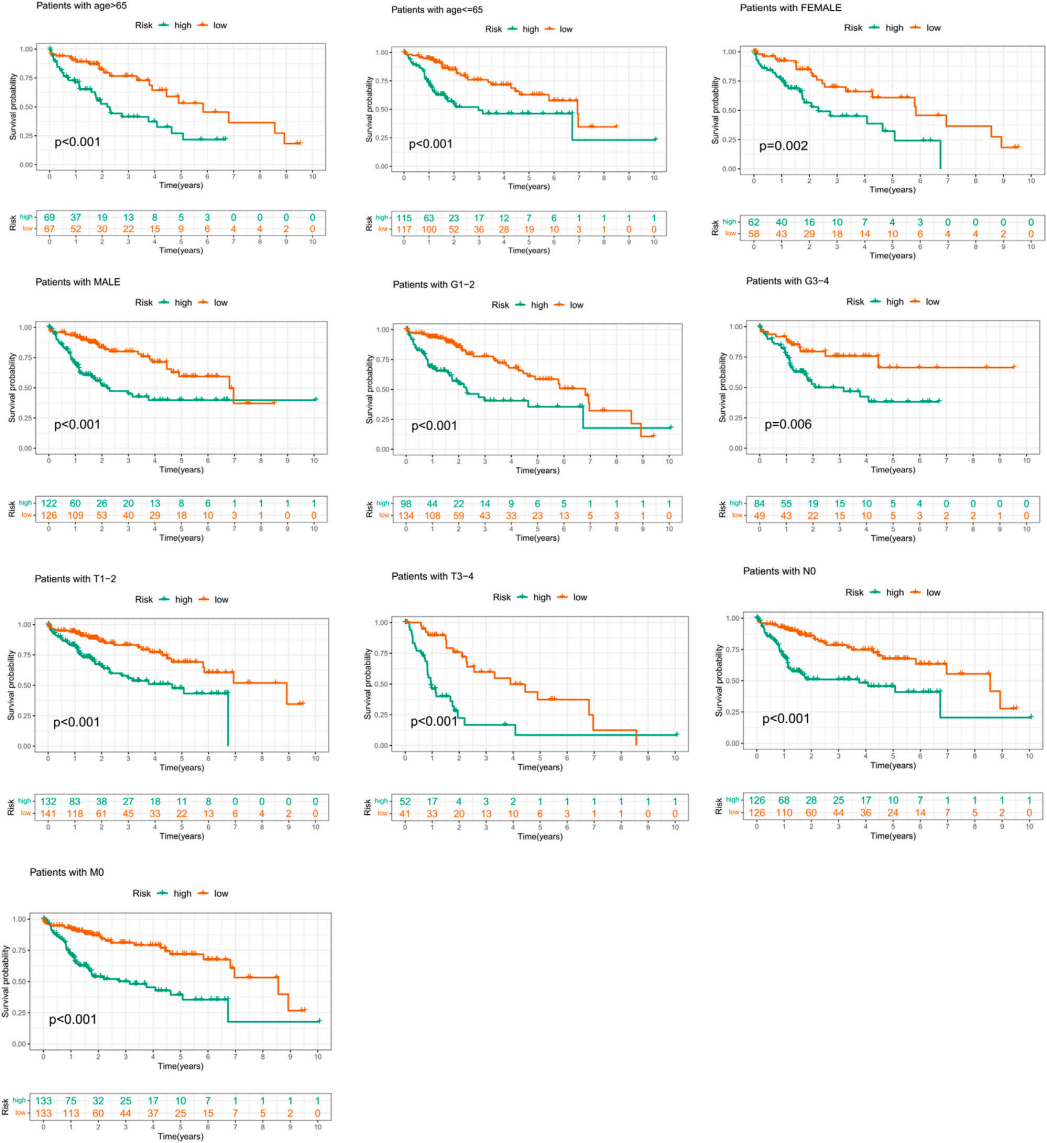
Comparison of survival of patients by risk signature score within each subgroup.

In order to prove that the prognostic risk score was not subject to differences between populations, we conducted subgroup survival analysis of people with different clinical characteristics, including population of <65 years old, ≥65 years old, male, female, Grade1–2, Grade3–4, T1–2 stage, T3–4 stage, N0, and M0 stage. Considering that the sample size of “M1” and “N1” subgroups was too small to be representative, the two subgroups have not been further validated. Because of the majority of TNM staging (AJCC) focused on the T status, the population of “stage I–II” and “stage III–IV” was not integrated into the study. In our study, only four patients had M0 stage disease and four patients with N0 stage, 233 patients were diagnosed as non-metastasis (N0M0), and the two subgroups largely overlapped. To avoid repetitive results, only those patients with M0 disease were included in the survival analysis in our analysis. It was worth noting that the OS of the low-risk group was better than that of the high-risk group (Figure 6). These results suggested that the suitability of prognostic risk model was not limited by population differences.

### Correlation between clinicopathological features and risk score

**FIGURE 7:**
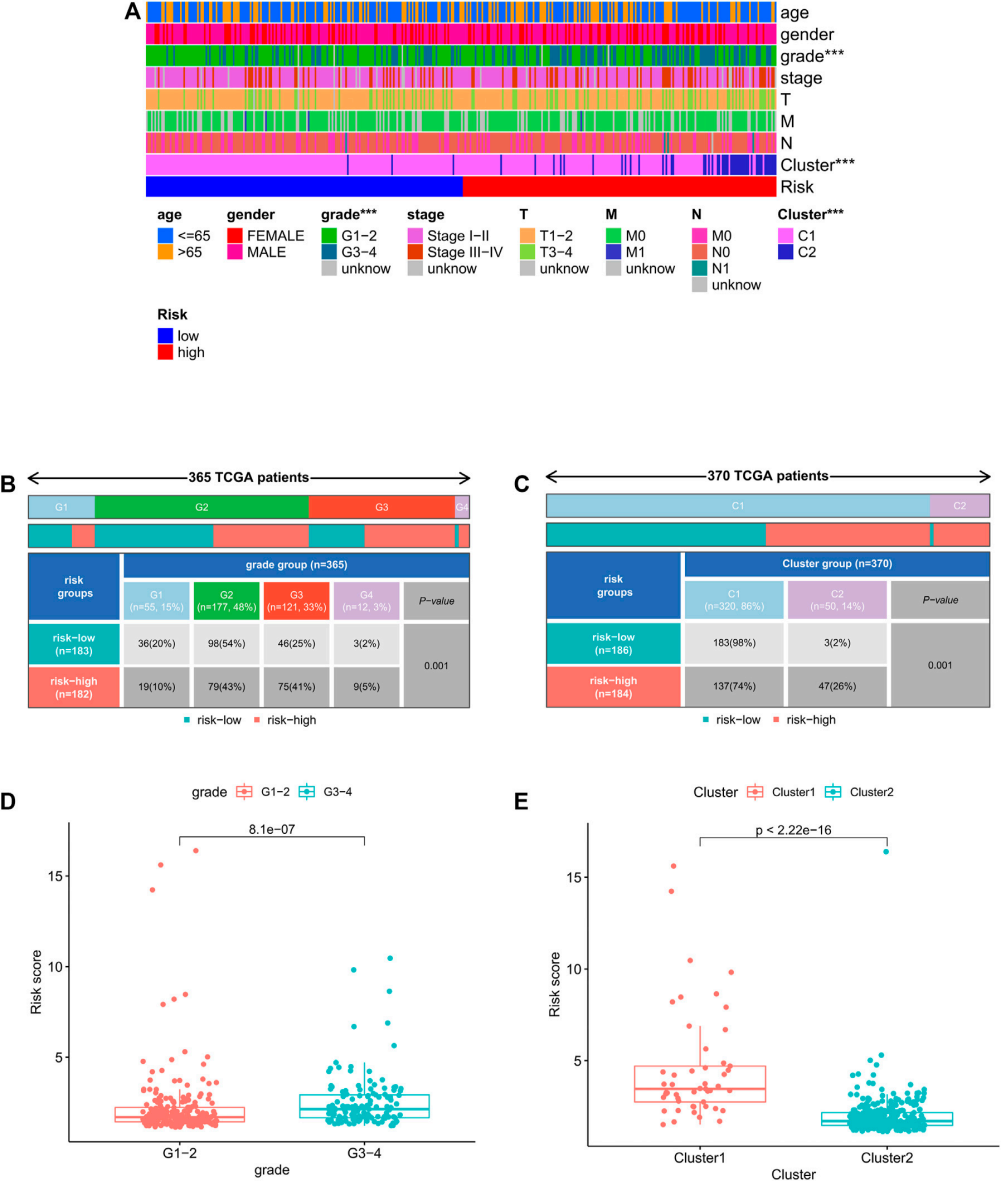
Relationship between risk score and clinicopathologic features. **(A)** Heatmap of relationship between risk scores and clinicopathologic features. *p < 0.05, **p < 0.01, and ***p < 0.001. **(B–C)** Distribution of risk scores stratified by grade and cluster. **(D)** Comparison of risk score distributions between G1–2 and G3–4. **(E)** Comparison of risk score distributions between cluster1 and cluster 2.

We further analyzed the correlation between clinicopathological features and prognostic risk score in the whole cohort. The results showed that risk score is significantly related to histological grade and clustering classification (Figure 7A). Furthermore, patients with grade 3/4 or cluster 1 tend to have higher risk score (Figure 7B–E).

### The nomogram system improved the prognostic risk score model

**FIGURE 8:**
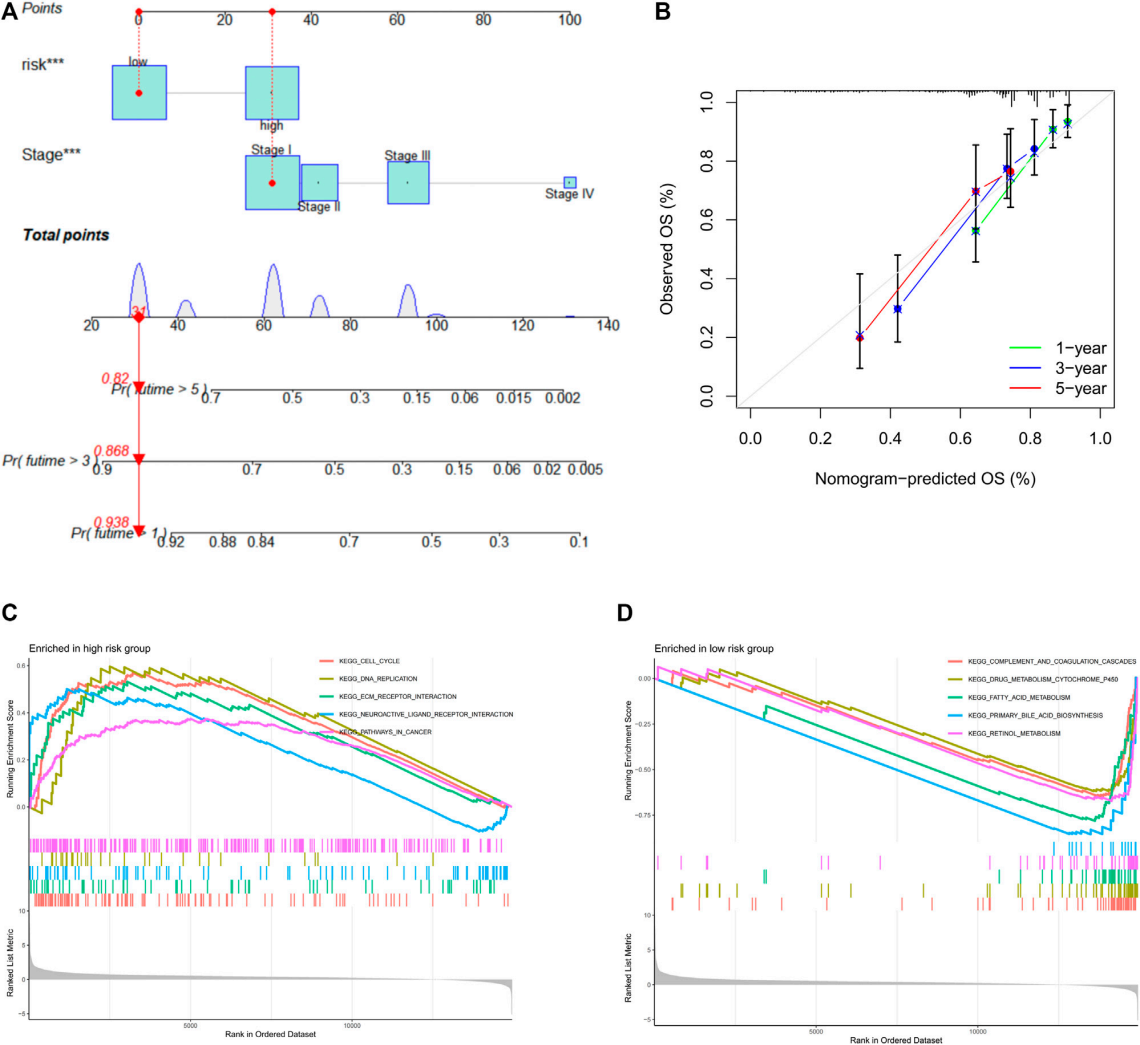
Nomogram prognostic model. **(A)** Nomogram constituting stage and risk score for predicting 3- and 5-year overall survival probability. **(B)** Calibration curves for the performance evaluation of the nomogram prognostic model. *X*-axis: nomogram predicts OS; *Y*-axis: actual OS. **(C)** GSEA results of the high-risk group. **(D)** GSEA results of the low-risk group. GSEA: Gene Set Enrichment Analysis.

To establish a quantitative method for predicting the survival probability of HCC patients, we integrated risk score and pathological stage to develop a nomogram to predict the survival probability of the 1-, 3-, and 5-year OS in the TCGA cohort (Figure 8A). Based on the nomogram, the survival for patients was predicted by calculating the set points based on each nomogram score. The performance of the nomogram was evaluated by Harrell’s concordance index (C-index). The C-index value for the nomogram calculated by “coxph” function of the R package “package” was 0.721 (95% CI: 0.656–0.786). Calibration plots suggested that our nomogram performed well (Figure 8B).

### Significant enrichment pathways of high- and low-risk groups

To explore the potential molecular mechanisms of prognostic risk signature, Gene Set Enrichment Analysis (GSEA) was performed in high-risk and low-risk groups, respectively. The top five significantly enriched pathways were displayed, GSEA indicated that the high-risk group was highly enriched in cell cycle signaling pathway, DNA replication signaling pathway, tumor signaling pathway, ECM receptor interaction signaling pathway, and neuroactive ligand receptor signaling pathway (Figure 8C), whereas the low-risk group was mainly enriched in metabolic-related pathways (Figure 8D). In conclusion, GSEA results showed that high-risk patients preferred to carcinogenesis, which also provided favorable evidence for the poorer prognosis of patients in the high-risk group than the low-risk group.

### Analysis of tumor immune microenvironment and tumor immune cell infiltration

**FIGURE 9:**
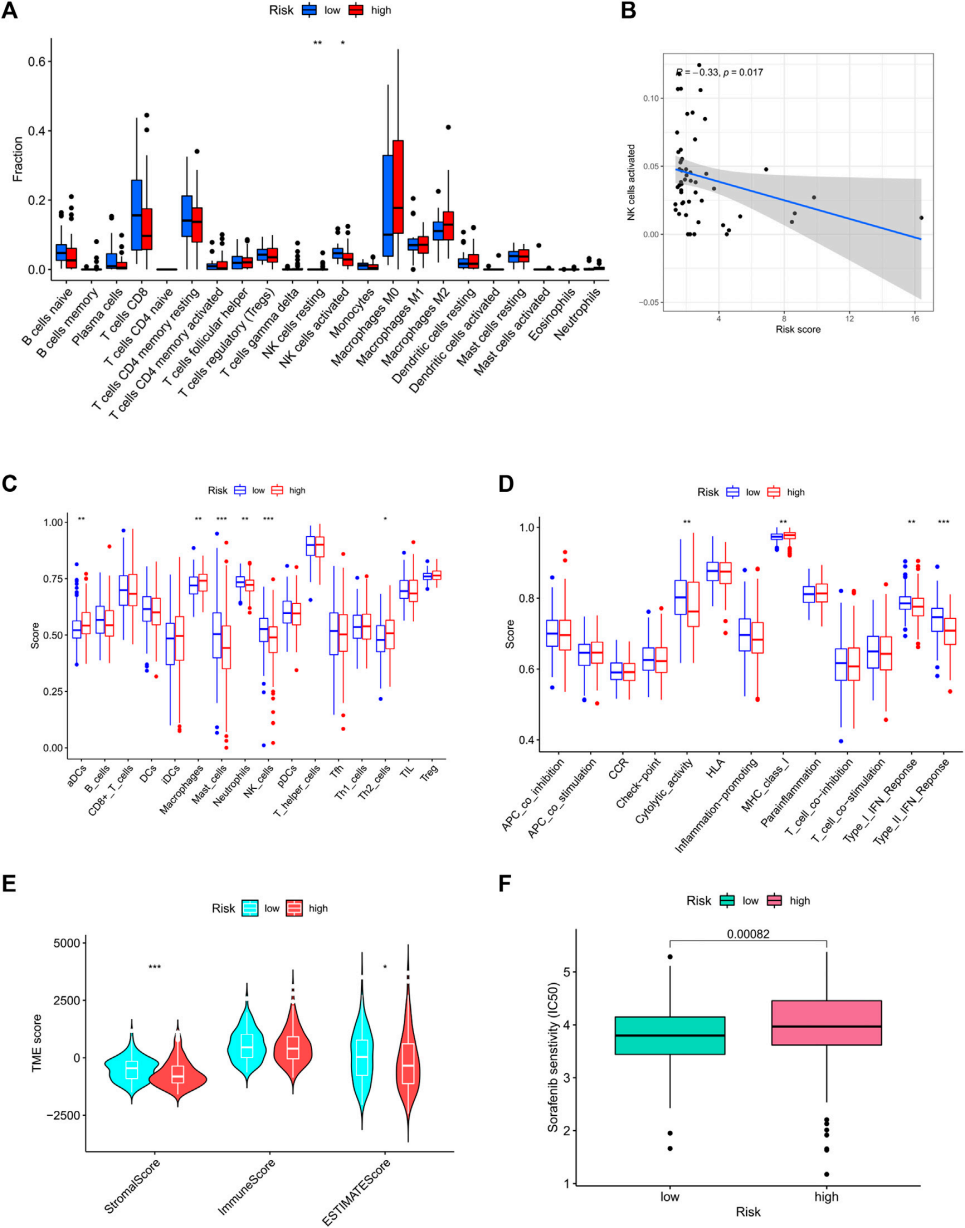
Immune cell infiltration in high- and low-risk groups. **(A)** The infiltrating levels of 22 immune cell types. **(B)** Correlation of NK cell level with risk score. **(C)** ssGSEA result of immune cell populations. **(D)** ssGSEA result of immune function. **(E)** Analysis of tumor microenvironment in high- and low-risk groups; Drug sensitivity result. **(F)** IC50 value of sorafenib in high- and low-risk groups. ssGSEA: Single-sample gene set enrichment analysis; IC50: Semi-inhibitory concentration.

The relationship between risk score and the infiltration levels of immune cells was predicted by evaluating the infiltration levels of 22 immune cells in the high/low risk groups. The results showed that compared with the low-risk group, the activated natural killer (NK) cell infiltration in the high-risk group was lower than that in the low-risk group (Figure 9A). After further analyzing the correlation between the risk score and NK cell infiltration, we found a negative correlation between the two (*R* = − 0.33, *p* < 0.05, Figure 9B). At the same time, single sample gene set enrichment analysis (ssGSEA) was applied to the RNA sequencing data of liver cancer tissues derived from TCGA to explore the differences of 16 kinds of immune cells and 13 kinds of immune functions in the high- and low-risk groups. The results showed that the levels of aDCs and macrophages infiltration in the high-risk group were significantly higher than those in the low-risk group, the infiltration levels of mast cells, neutrophils, and NK cells in the low-risk group were significantly higher than those in the high-risk group (Figure 9C). In terms of immune function, the scores of cytolytic activity (CYT), type I interferon response, and type II interferon response in the low-risk group were significantly higher than the high-risk group (*p* < 0.01), whereas the major histocompatibility antigen complex I score in the high-risk group was higher than that in the low-risk group (*p* < 0.01) (Figure 9D). Tumor microenvironment plays an important role in the development of tumors, including tumor cells, immune cells, stromal cells, extracellular matrix and so on. In this study, the ESTIMATE algorithm was used to quantify the ratio of stromal cells to immune cells in tumor samples from high- and low-risk groups. “Stromal score” represents stromal cells in tumor tissue and “immune Score” represents immune cells in tumor tissue. The results showed that in the low-risk group, the scores of stromal cells were significantly higher than immunocytes (Figure 9E). Although there was no statistical difference in the immune cell scores between the high-risk group and the low-risk group, the comprehensive scores of stromal cells and immune cells in the low-risk group were higher than the high-risk group.

### Patients with high risk were insensitive to sorafenib

To determine whether risk score affected tolerance to sorafenib treatment and if the screened patients could benefit from sorafenib treatment, we determined the value of IC50 in each group. Results suggested that patients with high risk were less sensitive to sorafenib than patients of the low-risk group (Figure 9F).

### The prognostic model predicted differential expression of immune checkpoint

**FIGURE 10:**
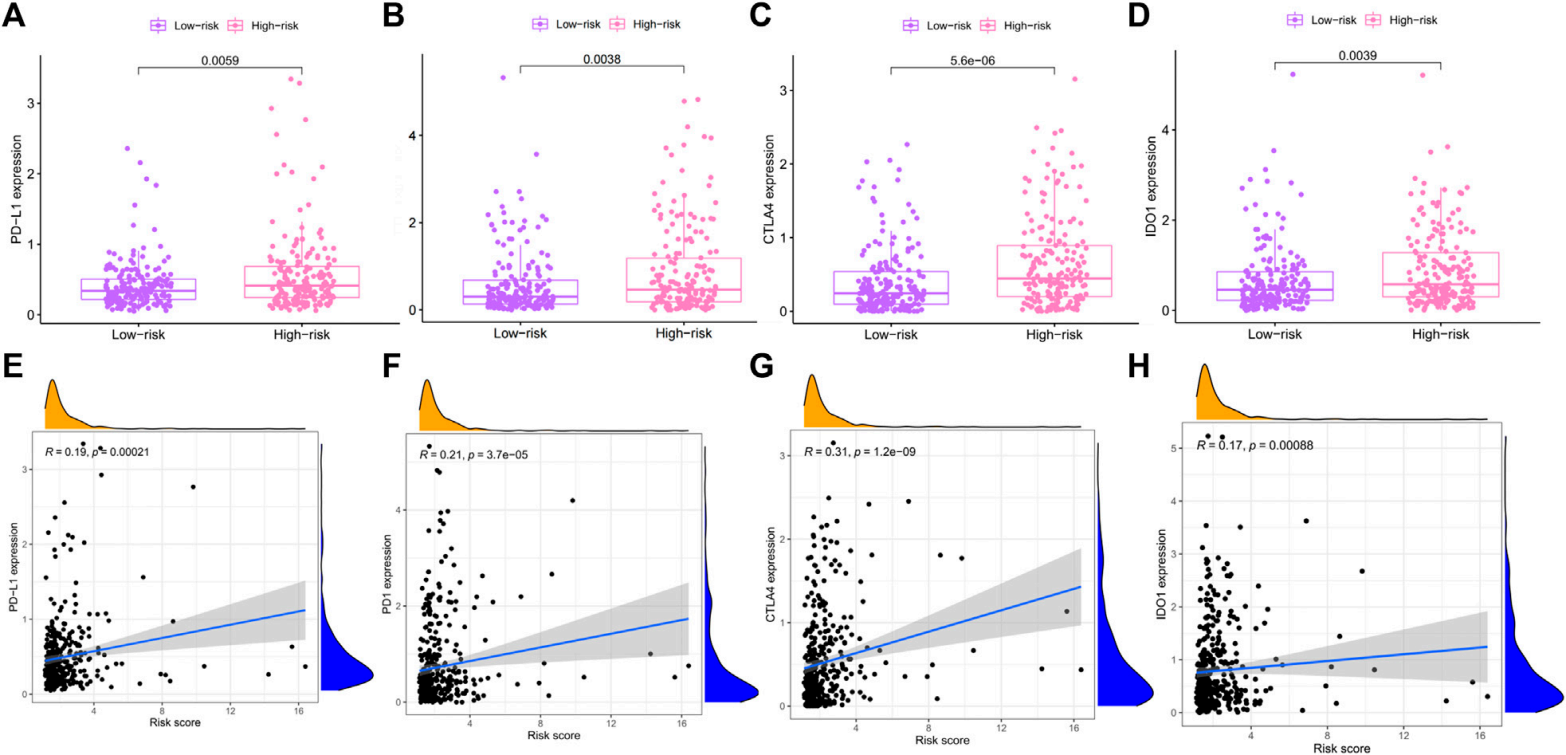
Association between risk score and immune checkpoint. **(A–D)** The differential expression of PD-L1, PD-1, CTLA4, and IOD-1 in high- and low-risk groups. **(E–H)** Correlation between PD-L1, PD-1, CTLA4, and IOD-1 and risk score were evaluated by Pearson correlation analysis. PD-1: Programmed Death receptor 1; PD-L1: Programmed Death Ligand 1; CTLA4: Cytotoxic T Lymphocyte Antigen 4; IOD-1: Indoleamine-1.

At present, immunotherapy has brought the dawn to liver cancers. The expression of immune checkpoint molecules is an important basis for determining whether patients could benefit from immunotherapy. In this study, we evaluated the expression levels of four key immune checkpoint molecules in high- and low-risk groups. As presented in Figure 10A–D, the expressions of PD1, PD-L1, CTLA4, and IDO-1 in the high-risk group were significantly higher than the low-risk group. In addition, risk score was positively associated with the expression of PD1 (r = 0.3; *p* < 0.001), PD-L1 (R = 0.27; *p* < 0.001), CTLA4 (R = 0.33; *p* < 0.001), and IDO-1 (r = 0.17; *p* < 0.001). The higher the risk score, the higher the expression of these four immune checkpoints.

### The differential expression of PRlncRNAs in cell lines and HCC tissues

**FIGURE 11:**
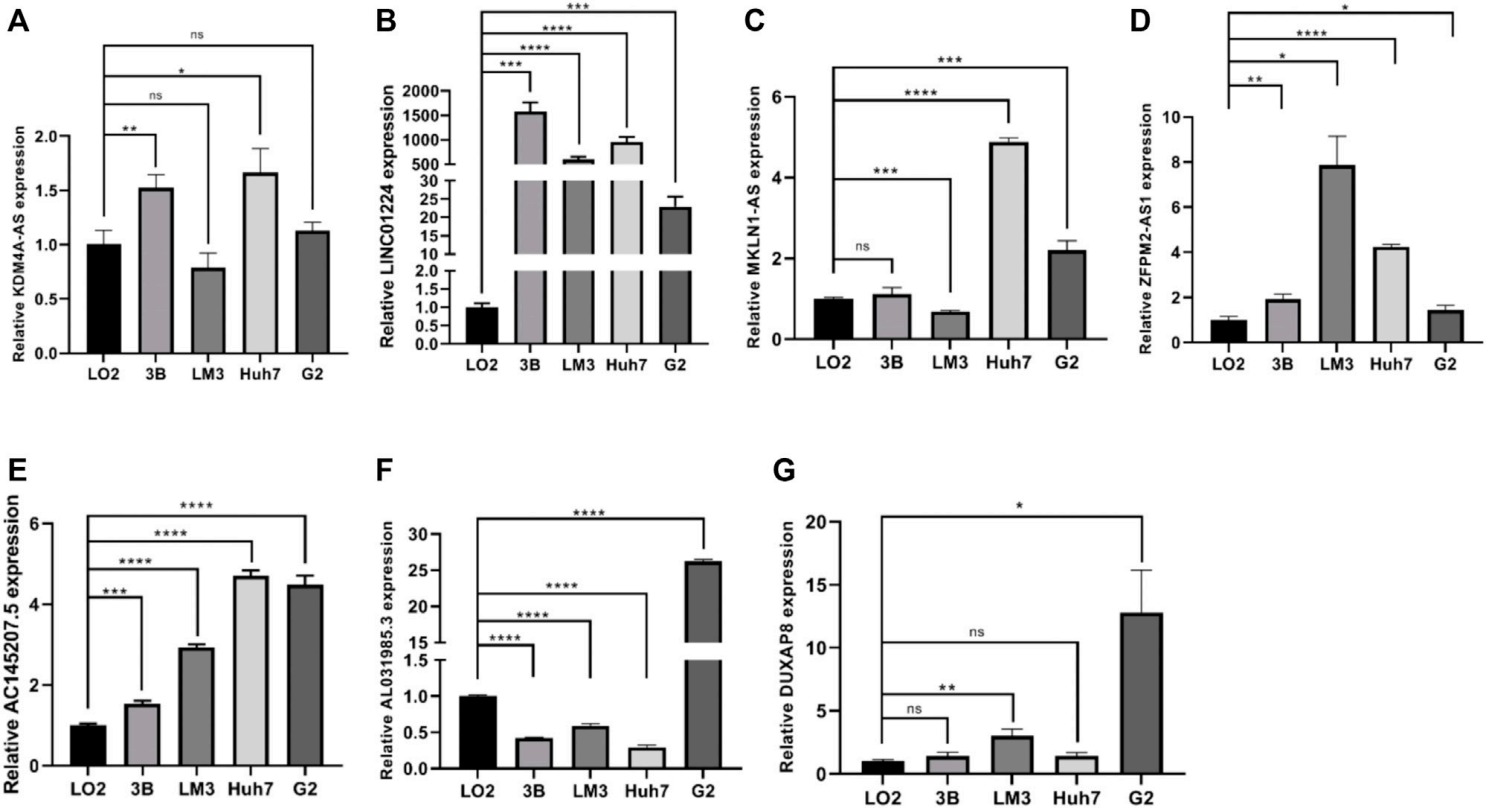
**(A–G)** The expression differences of KDM4A-AS1, LINC01224, MKLN1-AS, ZFPM2-AS1, AC145207.5, AL031985.3, and DUXAP8 in normal liver tissue and HCC tissue.

In the preliminary work, a prognostic model based on 10 necroptosis-related lncRNA was constructed, internal validation method was used to validate this model. Now, we further verified these results *in vitro*, seven lncRNAs of the model were subjected to quantitative reverse transcription PCR (qRT-PCR). Compared with LO2 cell, the expression levels of seven lncRNAs in four types of HCC cells were significantly differential (Figure 11A–G). Based on the TCGA database, we further verified the expression differences of AL031985.3, SREBF2-AS1, ZFPM2-AS1, KDM4A-AS1, AC026412.3, AC145207.5, DUXAP8, LINC01224, AC099850.4, and MKLN1-AS in normal liver tissue and HCC tissue. As shown in the results, the expression levels of these 10 lncRNA in liver cancer were significantly increased compared with normal liver tissue (Supplementary Figure S1).

## Discussion

Liver cancer is one of the most common causes of cancer death in the world. Most of the HCC cases are formed in the context of chronic viral liver diseases. Despite the development of effective antiviral therapy, liver cancer continues to rise. Non-alcoholic fatty liver disease (NAFLD), alcohol-related liver disease, and hereditary hemochromatosis are closely related to the incidence of liver cancer. The global burden of HCC is increasing, although about 40% of patients with primary cancer can be diagnosed early due to primary prevention, but after resection of the radical treatment, both surgery and liver transplantation, the residual liver is still prone to recurrence. As a result, almost half of liver cancer patients will eventually receive systemic treatment. Sorafenib is a kind of tyrosine kinase approved for patients diagnosed with advanced liver cancer and recommended as a standard first-line systematic treatment for patients with Child–Pugh A grade and BCLC-C (Llovet et al., 2008). In recent years, lenvatinib, the new first-line targeted drug, has been approved by FDA (Kudo et al., 2018), regorafenib (Bruix et al., 2017), cabozantinib (Abou-Alfa et al., 2018), and ramucirumab (Zhu et al., 2019) of the second-line drugs have been shown to improve clinical prognosis, although the median survival time is about 1 year. Therefore, we still need to find a breakthrough in the treatment of advanced liver cancer. In recent years, the field of immunotherapy has developed rapidly. Immune checkpoint inhibitors could kill tumors and prolong the prognosis of patients by awakening the immune system to produce anti-tumor immune response against progressive tumors, especially if a significant effect has been achieved in metastatic melanoma immunotherapy (doi: 10.1016/S1470-2045(0970334-1), Epub 2009 December 8).

The laboratory test including serum biomarkers, histochemical biomarkers, and other biomarkers are of great significance for early diagnosis, treatment decision-making, and prognosis prediction of HCC patients. Nowadays, AFP, AFP-L3, and DCP have become the most widely used serum biomarkers for early diagnosis of liver cancer (Yamamoto et al., 2010). AFP plays a guide role in the diagnosis of liver cancer, but its sensitivity and specificity are poor (Tzartzeva et al., 2018). In recent years, many scholars have devoted themselves to looking for new potential molecular markers. A previous study found that TP53 gene mutation is related to vascular invasion, poor prognosis, angiogenesis, metastasis, and drug resistance (Calderaro et al., 2017). The high expression of TERT can be used as an important prognostic index for intrahepatic metastasis of advanced hepatocellular carcinoma after radical resection (Yu et al., 2017). Biomarkers are helpful for guiding individualized targeted therapy, which could improve prognosis. Sorafenib resistance is frequently encountered in clinical practice, but there is still lack of suitable and validated biomarkers. Therefore, development of new biomarkers has important implications for clinical practice.

Previous studies have shown that RIPK3, RIPK1, and MLKL are the most important factors involved in necroptotic, playing important roles during cancer progression (Gong et al., 2019; Tan et al., 2020; Wu et al., 2020; Martens et al., 2021). It was found that the downregulated expression of RIPK3 occurs in M1 macrophages and facilitates tumorigenesis (Wu et al., 2020). RIPK1 leads to excessive activation of Caspase-8 and promotes hepatocellular carcinoma progression (Tan et al., 2020). Necrosome formed by RIPK1, RIPK3 and MLKL causing cells are susceptible to necroptosis by enhancing reactive production of oxygen species (Weinlich et al., 2017; Gong et al., 2019). In addition, it has been proved that some drugs inhibited the progression of liver cancer through PIPK1/PIPK3/MLKL signal. For example, triptolide (TPL) loaded into MP-LP has been proved to inhibit tumor growth effectively (Zheng et al., 2021). In summary, necroptotic affects the occurrence and development of liver cancer, especially necroptotic-related genes can serve as potential prognosis predictive biomarkers for HCC patients. Nowadays, some studies focus on the construction of prognosis model based on lncRNAs with different characteristics. For example, ferroptosis-related lncRNAs were associated with the prognosis and immune landscape of HCC patients (Xu et al., 2021). A prognostic model constructed by seven immune-related lncRNAs could be used as an independent prognostic evaluation index for HCC patients. Chen G. et al. (2021) identified three autophagy-associated lncRNAs (MIR210HG, AC099850.3, and CYTOR) as poor prognostic factors for HCC, demonstrating a shorter OS in high-risk groups. LncRNA-related prognostic models have been piled up in the literature, but there are no researches on necroptotic-related lncRNAs in HCC. Therefore, we constructed a necroptotic-related lncRNA prognostic model in this study, aimed to explore prognostic factors and a novel model to better guide immunotherapy and optimize patient outcomes.

In this study, we downloaded liver cancer transcriptome data and clinical data from TCGA database, converted the transcriptome data into TPM format by R language, and calculated the expression of each sequenced gene in each sample. In our study, 58 DEGs affected prognosis were significantly obtained by univariate Cox regression analysis. Then, we applied Lasso-Cox method to filter the collinear factors and obtained 10 candidate lncRNAs (AL031985.3, SREBF2-AS1, ZFPM2-AS1, KDM4A-AS1, AC026412.3, AC145207.5, DUXAP8, LINC01224, AC099850.4, and MKLN1-AS), from which the prognostic risk model was constructed. All the samples were randomly divided into experimental group and verification group, then each group was divided into high-risk subgroup and low-risk subgroup. By comparing the OS of patients in the high-risk group and the low-risk group, we found that the patients with high-risk score exhibited a shorter survival rate. The internal verification group verified the results again, we concluded that the risk score is a poor prognostic factor for HCC patients. In the clinical correlation analysis, risk score was significantly correlated with histological grade and cluster classification. ROC analysis further revealed that the risk signature we constructed could function as a sensitive indicator predicting 1-, 2-, and 3-year survival rates for the HCC patients. At the same time, we further compared the predictive effectiveness of the prognostic risk score with other clinical prognostic factors, we can see that among selected candidate factors, risk score performed optimally. At the same time, results of multivariate Cox regression analysis proved that the risk score is an independent risk factor in predicting the prognosis of HCC patients. Among all candidate lncRNAs in our model, AL031985.3 and AC145207.5 have been proved to play an important role in glycolysis-related prognostic models of liver cancer (Xia et al., 2021). The value of SREBF2-AS1 in HCC remains to be confirmed, but a study has found it can be used as ferroptosis-related lncRNA to predict the prognosis and immune activity of HCC (Chen Z. A. et al., 2021). ZFPM2-AS1 is highly expressed in HCC, which promotes the proliferation and migration of HCC cells by up-regulating hypoxia-inducible factor (HIP-1a) (Song et al., 2021). KDM4A-AS1 promotes HCC progression by activating the AKT pathway to promote the expression of a2KPNA2 (Chen T. et al., 2021). AC145207.5, as an immune-related lncRNA, could predict tumor immune infiltration cell and response to immunotherapy in HCC patients (Zhou et al., 2021). Hu et al. (2020) found that DUXAP8 promotes varieties of malignant phenotypes in HCC and resistance to PARP inhibitors by up-regulating FOXM1. There is growing experimental evidence that LINC01224 plays a role in promoting tumor progression in melanoma and colon cancer and shows resistance to radiotherapy (Chen L. et al., 2021; Cui et al., 2022). MKLN1-AS acts as an endogenous sponge of miR-654-3p to promote the progression of HCC (Gao et al., 2020). There is a paucity of studies with AC026412.3 to date, relevant researches about AC026412.3 need further excavation. Most of existing studies on the prognosis lncRNAs we obtained are remaind at the level of prognostic model, there are few studies have further explored the mechanism of these genes *in vivo* and *in vitro*, hence those lncRNAs may be clinically valuable and are worth further studied. Hence, the studies of the biological function and potential mechanism of lncRNAs in liver cancer are extremely important for the exploration of new therapeutic targets for liver cancer.

In terms of mechanism, in order to further explain the mechanism of DEGs, all DEGs were subjected to GO and KEGG enrichment analyses. Firstly, results revealed that the biological functions of the majority of the DEGs are closely related to the process of cell cycle, including organelle division, mitosis, and chromosome segregation. Secondly, chromosome, microtubule, and spindle structure were the most dominant terms in the cellular component. While “ATP enzyme activity,” “small GTP enzyme binding activity,” and “RASGTP enzyme binding activity” were the most abundant terms in the molecular function category. From the above results, it can be concluded that DEGs play an important role in the process of cell division. We do know that the occurrence and development of tumors are usually related to the abnormal accumulation of genes, these abnormal mutations usually occur when cell cycle processes were disrupted (Stratton et al., 2009; Suski et al., 2021). The results of KEGG analysis suggested that DEGs are mainly enriched in viral infection-related pathways, especially the pathways of “herpes simplex virus,” “human papillomavirus,” and “coronavirus-COVID-19,” which have attracted worldwide attention in the past 3 years. It is well known that hepatitis B virus infection is a main risk factor of HCC in China (Levrero and Zucman-Rossi, 2016), so it can be speculated that DEGs may be involved in the process of “hepatitis B-liver cirrhosis-liver cancer trilogy.” At present, few people studied the role of other viruses in the occurrence and development of liver cancer. HPV disrupts the normal cell cycle and promotes the accumulation of genetic damage, leading to tumorigenesis (Araldi et al., 2018; Szymonowicz and Chen, 2020). Previous studies have confirmed that persistent infection of papillomavirus will lead to the occurrence of cervical cancer (Araldi et al., 2018). As with hepatitis B virus, human papillomavirus is a DNA virus, which integrated into the host genome after invading human body (Szymonowicz and Chen, 2020). The results of KEGG gave us new enlightenments: DEGs may be involved in the viral infection pathways and this process may occur to hepatocarcinogenesis.

Liver is an organ that plays a dual role of metabolism and immunoregulatory. There are a large number of immune cells in the liver, including macrophages (Kupffer cells), liver sinusoidal endothelial cells (LSECs), and natural killer cells (Jenne and Kubes, 2013), LSECs act as antigen-presenting cells (APCs) along with Kupffer cells and dendritic cells (DCs) (Thomson and Knolle, 2010). In addition, the liver contains a large population of liver-resident lymphocytes, encompassing NK cells and T cells, exerting innate immune responses against viruses, intracellular bacteria, tumors, and parasites (Gao et al., 2008), which play a protective role in the liver by exerting cytotoxic activity. Tumor microenvironment is a complex and constant evolution, in addition to stromal cells, fibroblasts, and endothelial cells, TME also includes innate and acquired immune cells. Increasing evidence suggests that innate immune cells and acquired immune cells promote tumor progression in tumor microenvironment. Studies have shown that “hot tumors” recognize and attack tumors by abundant CD4^+^ and CD8^+^ tumor infiltrating lymphocytes, which are generally associated with better prognosis as well as improved response to ICB (Chen and Mellman, 2017). Therefore, the level of immune infiltrating cells in tumor microenvironment has implications for host anti-tumor response and immunotherapy. In this study, we explored the differences in the level of immune infiltrating cells between high- and low-risk groups. The level of NK cells infiltrated in the high-risk group was significantly lower than that in the low-risk group. By further analyzing the correlation between the level of NK cell and risk score, it was found that there was a significant negative correlation. NK cells are known to play a key role in innate immune surveillance against tumors. NK cells account for half of the number of liver lymphocytes, they are cytotoxic killer cells with the function of anti-tumor, leading to an efficient antitumor activity mediated by releasing of cytotoxic granules, TRAIL, and Fas-L (Robinson et al., 2016). Such studies have shown that exocrine circUHRF1 inhibits the function of NK cells by degrading miR-449c-5p and up-regulating the expression of TIM-3, resulting in drug resistance to anti-PD-1 immunotherapy in HCC patients (Zhang et al., 2020). In our study, patients with high risk score to be the lower level of NK cell infiltration. Therefore, we speculated that patients in the high-risk group presented with weak immune surveillance ability, triggering the initiation of cancer in susceptible individuals and be more likely to develop drug resistance to treatment. Other studies have shown that approximately 25% of HCC patients with a high inflammatory score showed high or moderate levels of lymphocyte infiltration (Sia et al., 2017). Tumor infiltrating lymphocytes (TILs) constituted the main component of solid tumor microenvironment and mediated anti-tumor response (Qin, 2012). However, such cellular response could be dysfunctional due to the increased CD4^+^/CD8^+^ T cells, which leads to immune tolerance and confers an even poorer prognosis (Fu et al., 2007). Similar results were observed in our analysis: The infiltration levels of activated dendritic cells, tumor-associated macrophages, and Th2 cells in the high-risk group were significantly higher than the low-risk group, while the infiltration levels of mast cells, neutrophils, and NK cells in the low-risk group were significantly higher than the high-risk group. It has been found that chemotherapy-induced immunogenic cell death results in the release of stimulators, enhancing DC subsets cross-present antigen to CD8 + T cells, and thus augmenting CD8 + T cell responses (Sánchez-Paulete et al., 2017). Moreover, a recent report showed that the infiltration level of DC is associated with the ICB-based immunotherapy (Barry et al., 2018). At first, it is believed that macrophages played an anti-tumor effect. After years of clinical trials and fundamental studies, it has been proved that macrophages promote cancer progression in most of the cases. In a meta-analysis, more than 80% of studies found that the density of M2 phenotype macrophages is correlated with poor prognosis (Bingle et al., 2002). Neutrophils reflect the inflammatory state of the host, which is one of the hallmarks of cancer (Hanahan and Weinberg, 2011). The high expression of neutrophils is related to the poor prognosis of several solid tumors, some researchers believe that neutrophils promote the occurrence and development of tumors by releasing reactive oxygen species (ROS) and reactive nitrogen species(RNS) (Antonio et al., 2015), they also facilitate tumor cell metastasis by inhibiting function of natural killer and promoting tumor cell extravasation (Welch et al., 1989; Spiegel et al., 2016). However, in a prospective clinical study, univariate COX regression analysis of potential prognostic factors showed that neutrophils are significantly associated with superior overall survival in gastric cancer subjects (Caruso et al., 2002). In conclusion, we evaluated immune cells in the tumor microenvironment of patients with different risk scores, our result has important practical implications in predicting prognosis and therapeutic.

In the course of cancer occurrence and development, with the evolution of the tumor, tumor cells have acquired a variety of mechanisms to evade immune surveillance and inhibited anti-tumor immune response to escape attacks from the host immune system. Immune checkpoint inhibitors have brought considerable clinical benefits to patients and promoted the development of oncology greatly. Despite immune checkpoint inhibitors for the clinical treatment of cancer has led to durable responses for some patients, only a small fraction of patient response to immune checkpoint inhibitors. There are many factors that are involved in the efficacy of immune checkpoint inhibitors treatments, including infiltration of cytotoxic T cells (Van Allen et al., 2015), neoantigen load and mutation frequency (Snyder et al., 2014), serum PD-L1 levels (Nishino et al., 2017), and mismatch repair deficiency (Le et al., 2015). Indoleamine 2,3 dioxygenase 1 (IDO1) as an immune checkpoint molecule converts tryptophan to kynurenine, which is associated with tumor immunosuppression. In a phase II clinical study, it was found that most sarcoma cells were infiltrated by IDO1-expressing M2 macrophages, resulting in a low response to PD-(L) 1 inhibitor (Munn and Mellor, 2016; Li et al., 2017; Toulmonde et al., 2018). However, none of these factors are sufficient to achieve accurate prediction. At present, identification of immunotherapy biomarkers and regulators modulating resistance is a critical challenge in this field. In this study, we analyzed the expression levels of four immune checkpoint molecules in high- and low-risk groups. The results showed that the expression of immune checkpoint molecules in the high-risk group were higher than that in the low-risk group. High expression of immune checkpoints is usually associated with immune escape and drug resistance (Peng et al., 2019; Lv et al., 2021), suggesting that patients in the high-risk group might be more likely to immune escape and drug resistance, and may not be suitable for immunotherapy. The treatment options of patients with advanced liver cancer are limited, immunotherapy may bring hope to some patients, but our study found that patients with high-risk are more likely to develop drug resistance, which speculated that the prognosis of patients with high-risk is worse.

In the end, we analyze lncRNA expression in normal hepatic and HCC cells to verify the accuracy of our model. Our RT-qPCR data showing that the expressions of seven lncRNAs were significant different in HCC cells compared to the normal hepatic cells. All these data suggested that our signature has a crucial role in HCC development and progression. All in all, this work has developed a prognostic prediction model for necroptosis-related lncRNAs by analyzing data from the TCGA public database, providing references for patients’ prognosis and clinical guidance, the limitations of this study should be considered. Although results were validated in the TCGA cohort, its reliability needs to be further verified in other independent cohorts. In addition, the ability of the necroptosis-related lncRNAs improving the efficacy of immunotherapy in HCC patients has not been proved, we need to further confirm it by *in vivo* and *in vitro* experiments in the future. Secondly, expression levels of necroptosis-related lncRNAs in biological specimens have not been clearly validated. Finally, in the future, we need to further explore the potential mechanism behind the prognostic model affecting the process of HCC, so as to provide a new target and therapy for the clinical treatment of HCC, improving systemic treatment efficacy and thus prolong the overall survival time of HCC patients.

## Data Availability

The original contributions presented in the study are included in the article/Supplementary Material. Further inquiries can be directed to the corresponding author.
